# A Novel Approach to Evaluating the Iron and Folate Status of Women of Reproductive Age in Uzbekistan after 3 Years of Flour Fortification with Micronutrients

**DOI:** 10.1371/journal.pone.0079726

**Published:** 2013-11-19

**Authors:** Lauren Hund, Christine A. Northrop-Clewes, Ronald Nazario, Dilora Suleymanova, Lusine Mirzoyan, Munira Irisova, Marcello Pagano, Joseph J. Valadez

**Affiliations:** 1 Harvard School of Public Health, Boston, Massachusetts, United States of America; 2 Global Alliance for Improved Nutrition (GAIN), Geneva, Switzerland; 3 Instituto de Investigación Nutricional, Lima, Perú; 4 Ministry of Health, Research Institute of Hematology and Blood Transfusion, Tashkent, Uzbekistan; 5 Joint Projects Implementation Bureau “Health-2” and “Woman and Child Health Development” Tashkent, Uzbekistan; 6 Liverpool School of Tropical Medicine, Liverpool, United Kingdom; 7 University of New Mexico, Albuquerque, United States of America; National Institute of Child Health and Human Development, United States of America

## Abstract

**Background:**

The Uzbekistan 1996 Demographic Health Survey reported 60.4% of women of reproductive age (WRA) had low hemoglobin concentrations (<120 g/L), and anemia was an important public health problem. Fortification of wheat flour was identified as an appropriate intervention to address anemia due to the ubiquitous consumption of wheat flour. A National Flour Fortification Program (NFFP) was implemented in 2005.

**Methodology/Principal Findings:**

After 3-years of the NFFP, a national survey using large country-lot quality assurance sampling was carried out to assess iron, folate, hemoglobin and inflammation status of WRA; the coverage and knowledge of the fortified first grade *UzDonMakhsulot* (UDM) flour/grey loaf program; and consumption habits of women to investigate the dietary factors associated with anemia. Estimated anemia prevalence was 34.4% (95% CI: 32.0, 36.7), iron depletion 47.5% (95% CI: 45.1, 49.9) and folate deficiency 28.8% (95% CI: 26.8, 30.8); the effect of inflammation was minimal (4% with CRP >5 mg/L). Severe anemia was more prevalent among folate deficient than iron depleted WRA. Presence of UDM first grade flour or the grey loaf was reported in 71.3% of households. Among WRA, 32.1% were aware of UDM fortification; only 3.7% mentioned the benefits of fortification and 12.5% understood causes of anemia. Consumption of heme iron-containing food (91%) and iron absorption enhancers (97%) was high, as was the consumption of iron absorption inhibitors (95%).

**Conclusions/Significance:**

The NFFP coincided with a substantial decline in the prevalence of anemia. Folate deficiency was a stronger predictor of severe anemia than iron depletion. However, the prevalence of iron depletion was high, suggesting that women are not eating enough iron or iron absorption is inhibited. Fortified products were prevalent throughout Uzbekistan, though UDM flour must be adequately fortified and monitored in the future. Knowledge of fortification and anemia was low, suggesting consumer education should be prioritized.

## Introduction

Iron-deficiency anemia (IDA) is a significant public health problem in Uzbekistan. In 1996, a Demographic Health Survey (DHS) reported 60.4% of women of reproductive age (WRA) and 60.8% of pre-school age children (pre-SAC), between 6-59 months, had low concentrations of hemoglobin (hemoglobin <120 g/L and <110 g/L respectively) [1]. In 2002, the Health Examination Survey reported that 49.2% of pre-SAC had hemoglobin concentrations below the World Health Organization (WHO) cut-off of <110 g/L; however, there were no data for WRA [2]. Recently, data on WRA were collected as part of the evaluation of the Program on Prevention and Control of Anemia in Uzbekistan in 2005 from Khoresm and Fergona oblasts and the Republic of Karakalpakstan. Using this survey, an estimated 37% of WRA had anemia [3]. There are no nationally representative data on the prevalence of folate deficiency. Using data from four sentinel sites between the years 2004 and 2007, 20% to 50% of non-pregnant women may have anemia and low levels of iron; and 24% to 34% may have low concentrations of folate (<10 nmol/L)[4].

Fortification of wheat flour was identified as an appropriate public health intervention to address anemia due to the ubiquitous consumption of wheat flour (Uzbekistan Nutritional Investment Plan, 2008). In urban areas, >90% of urban households buy bread. A separate supply chain ensures that industrial urban bakers are supplied with first grade, fortified flour for the fortified grey loaves. The pattern of bread consumption is very different in rural areas of Uzbekistan, where people bake bread at home or buy it from small ‘tandoor’ bakers; it is consequently a more difficult task to provide fortified bread to the rural poor [5]. In rural areas, most home-made bread is made from a mix of both first grade (fortified) and premium grade flour (non-fortified), which reduces the amount of fortified flour consumed and hence the micronutrient intake. In very poor areas, families may use their own home-grown flour, which is not fortified.

From 2001 to 2004, a program supported by the Asian Development Bank funded a pilot project in which 14 flour-mills in six provinces were equipped with fortification equipment (4). In 2005, the Republic of Uzbekistan's National Flour Fortification Program (NFFP) was implemented and executed by the Uzbekistan Ministry of Health and UNICEF. The NFFP was supported by the Global Alliance for Improved Nutrition (GAIN) and the World Bank, and received technical assistance from the US Centers for Disease Control and Prevention (CDC) and the Uzbekistan Institute of Hematology and Blood Transfusion. Building upon the Asian Development Bank project, funding from GAIN allowed expansion of production of fortified flour to an additional 34 mills. Uzbekistan produces four grades of flour: premium (extraction rate 55–60%), first (extraction rate up to 72%), second (extraction rate 78–83%) and third (extraction rate 83–95%). Since the first grade flour is consumed by 61% of the population, the NFFP targeted the fortification of ‘first grade’ flour produced by a large, state-run milling agency, *UzDonMakhsulot* (UDM), and some private mills, and on the ‘grey loaf’ made from that flour in commercial bakeries [6], [7].

The selected fortificant premix was the *KAP Complex 1*, which was developed by the Kazakhstan Academy of Nutrition and used in the Asian Development Bank regional flour fortification project [4]. The premix contains elemental iron powder made by an electrolytic process; consequently, the premix has twice the level of iron as fortificants made with ferrous sulphate, to compensate for a bioavailability of only 50%. In addition, the fortificant contains folic acid, zinc, thiamin, riboflavin, and niacinamide [4]. The KAP premix incorporation rate was 120 g/metric ton of flour, equivalent to 40 mg/kg of iron, providing an average of 22 – 39% of the WHO recommended nutrient intake (RNI) of iron (assuming consumption of 0.16 – 0.26 kg/day) [8]. The primary objective of the program was to reduce anemia among WRA by 20% (from 60% to 48%), using the 1996 DHS anemia data as a baseline [1].

At initiation in 2005, a monitoring and evaluation sentinel system was developed to monitor the health impact of the NFFP. Using the sentinel system, 40 households were randomly sampled within selected pilot regions [4]; this sample was not representative of all WRA in Uzbekistan. Therefore, a national Large Country – Lot Quality Assurance Sampling (LC-LQAS) survey was conducted in March/April 2008 to obtain nationally representative data on the iron and folate status among WRA in Uzbekistan. Additionally, the survey was used to inform about the success and uptake of the intervention. LC-LQAS is a survey design used to assess the impact of a national program by collecting small samples within clusters and is a practical tool for conducting end-line surveys.

In this paper, the results of the national survey are presented, assessing the iron, folate and inflammation status of non-pregnant WRA following the implementation of the NFFP. The prevalence of anemia, folate deficiency, and iron depletion are estimated, and the relationship between folate deficiency, iron depletion, and severity of anemia in this population is investigated. The success of the intervention implementation is assessed using coverage of the fortified flour and the knowledge about the fortification program among WRA. Consumption habits of the women are summarized to investigate the profile of dietary factors commonly associated with anemia in WRA in Uzbekistan, to understand other factors contributing to anemia in this population.

## Materials and Methods

### Ethics statement

Ethical permission was obtained from the Government of Uzbekistan with a Special Decree from the President of the Republic of Uzbekistan (No. 153, dated August 15, 2005) and an Order of the Ministry of Health of the Republic of Uzbekistan (No. 194 dated March 12, 2008). Inclusion in the survey was dependent on the women being willing to participate in the survey and giving written informed consent for both the interview and the phlebotomy.

The research team ensured that study participants were entered into the survey voluntarily, following the ethical principles summarized in the Belmont report (9). The informed consent form included the purpose of the survey, the steps involved, the length of interview, and the individual and public benefit of taking part. This form also emphasized the voluntary and confidential nature of the survey and made a clear statement informing each woman that she should make her own decision about participation. The women were given a chance to ask questions about the study. Those who agreed to take part signed the informed consent form and the interviewer also signed the form. The age range of eligible participants, 15 to 49 years, includes adolescents. Informed consent was administrated to adolescents in person, rather than to their parents or caretakers, based on the legislation of Republic of Uzbekistan which states: “Adolescents in the age of above fourteen years old have the right to give voluntary informed consent to undergo any medical intervention or to refuse it” (www.lex.uz/Pages/GetAct.aspx?lact_id=41329).

The results of the study were confidential and data were kept securely. The results of hemoglobin test were conveyed to the participants immediately. If the ferritin or folate concentrations were out of the normal range, an appropriate doctor was informed in order to contact the participant to follow up with any appropriate treatment.

### Survey design

In 2008, a national LC-LQAS survey was conducted to evaluate the performance of the Uzbekistan flour fortification program at national and oblast-level [6]. LC-LQAS surveys integrate lot quality assurance sampling (LQAS) and cluster sampling, resulting in a multi-level evaluation tool. In the LC-LQAS survey design, the cluster-level sample size is driven by the goal of local-level classification using the cluster data [9]. LQAS is a statistical tool used for the classification of the performance of small geographic or administrative units called supervision areas (SAs) [10]. The collection of rayons, cities, and towns within a region served as the SAs for the survey. This assessment is the first application of LC-LQAS to evaluate a fortification program.

### Sampling

Uzbekistan is divided into 14 regions: 12 oblasts, the Autonomous Republic Karakalpakstan and Tashkent city. Among the 14 regions in Uzbekistan, there are 162 rayons and 37 cities or towns, comprising 199 SAs. For the survey, the cities and towns were treated as separate administrative units from the rayons.

The number of sampled SAs per region was selected such that the maximum confidence interval width for coverage indicators at the oblast level was 20%. To calculate the sample size, population data were obtained directly from the census of the population. To anticipate the degree of clustering in the survey, the design effects were assessed from 28 nutritional and demographic variables in the Uzbekistan 2002 DHS, and the largest design effect, 1.79, was selected to calculate the sample size. Oblast-level sample sizes were calculated by using the relationship between the design effect and intra-class correlation, along with census population estimates, using the methods described in [9]. The number of WRA sampled per oblast was fixed at 19 to facilitate local level evaluation of the fortification program. To calculate the number of SAs to sample per oblast (n), the following formula was applied:
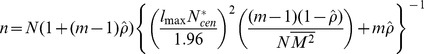
where *m*  = 19 is the number of WRA sampled per SA; *N* is the total number of SAs in the oblast; N^*^_cen_ the total population in the oblast; 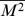 is the average of the square of the population in each SA; 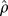
is an estimate of the intraclass correlation; and l_max_ = 0.2 is the maximum desired length for the confidence interval.

The specific SAs within each region were sampled using simple random sampling without replacement, following [9]. Within a SA, sampling locations were identified by listing the health points (rural medical centers known by the acronym SVP and urban policlinics) and their associated population size. Then, 19 random locations among the SVP/policlinics were selected using probability proportional to size (PPS) sampling. Within these locations, one household was randomly selected using segmentation sampling, and one WRA in the household was interviewed. If no WRA resided at the selected household, the data collectors proceeded to the next closest door. This PPS sampling strategy is self-weighting and approximates simple random sampling within an SA.

### Questionnaire

A survey questionnaire was developed to address the project's objectives. The questionnaire consisted of the following sections: 1) Introduction to the survey; 2) Demographic and socio-economic information; 3) Availability of fortified flour/bread at the household and testing of flour; 4) Purchase and consumption of fortified flour/bread at the household; 5) Knowledge of anemia and flour fortification; 6) Iron supplementation; 7) History of anemia; 8) Diet; 9) Reproductive history; and 10) Blood sampling for analysis of hemoglobin, serum folate, C- reactive protein (CRP) and ferritin. Before the start of the survey, two four-day parallel training sessions were conducted on the sampling method, data collection, and blood collection and storage. The questionnaire and blood sampling techniques were pre-tested in selected non-survey urban and rural locations, and results of the pre-test were incorporated into the final version of the instrument [11].

### Field work

Twenty field-teams were selected, comprising one interviewer and one laboratory worker. Ten supervisors were also appointed: seven provided direct supervisory activities for the interviewers (3 teams per supervisor) and the remaining three supervised the blood sampling and processing. Fieldwork took place from 17 March – 21 April, 2008.

### Blood collection

Venous blood (10 mL) was drawn from each WRA using a Vacutainer (BD, Russia) and kept in a cool and dark place until the end of each day. A small volume of the whole blood was used to measure the hemoglobin concentration to the nearest 1 g/L using a portable b–hemoglobin photometer (Hb 301 HemoCue™ AB, Angelholm, Sweden). Each HemoCue was checked three times a day using three control liquids (low, medium and high) and was considered acceptable for use only if the reading was within the range specified. Altimeters were used to measure the elevation above the sea level, to adjust hemoglobin concentrations where necessary (although few places in Uzbekistan are above 1000 meters). Hemoglobin concentrations were also adjusted for smoking status. Women with hemoglobin concentrations <120 g/L were classified as anemic (13). The remaining whole blood was centrifuged for 10 minutes at 3000 rpm; the serum was removed and stored cold (+4°C) in two tubes (one tube containing serum for the analysis of folate and one for CRP and ferritin) at local Blood Transfusion Center. At the end of each week, the serum was transported to the Hematology and Blood Transfusion Center, Tashkent where it was stored frozen at -70°C until analysis.

Serum CRP concentrations were measured using the Randox full-range immunoturbidometric assay (range of assay: 0.3 –161 mg/L) on a Randox Daytona Analyser (Randox Laboratories Ltd, Crumlin, UK). Three levels of specific protein controls and two high sensitivity CRP controls were run at the beginning and end of the day; intra-assay precision was <3% and inter-assay precision was <6%. A CRP concentration >5 mg/L indicated the presence of inflammation [12].

Serum ferritin concentrations were measured using the Randox immunoturbidometric assay (range of assay: 5.78–434 µg/L) also on the Randox Daytona Analyser. Three levels of specific protein controls were run twice a day; the intra-assay and inter-assay precision were <5%. A ferritin concentration <15 µg/L indicated depleted iron stores [13].

Serum samples were analyzed to determine folate concentrations, using a microbiological assay in the Department of Hematology at the Tashkent Hematology and Blood Transfusion Center. The microbiological assay was based on the methodology used by the Division of Laboratory Sciences, Nutritional Biomarker Branch at the CDC, USA, but with a slight modification because of the limitation of equipment available [14]. As it was not possible to read the plates at 590 nm, the plates were read at 492 nm. The use of the 492 nm wavelength was verified by CDC who found that compared to reading the plates at 592 nm, reading at 492 nm gave a bias of 1.6% (95% CI: 0.4 – 2.9%; SE 0.63%; n = 98), which was considered acceptable. Serum pools were prepared as quality control (QC) samples at three levels: high, medium and low, and were run at least 20 times to establish the acceptable ranges. For external QC, CDC serum pool samples were run on each plate and verified by CDC recurrently throughout the analysis period. The reportable range for serum samples is 10-100 nmol/L; serum folate concentrations <10 nmol/L were considered deficient.

### Data Analysis

The survey data were analyzed at the oblast and national level using Stata v12 (StataCorp LP© Lakeway Drive, College Station, Texas, USA). Large sample linearized variance estimates were used to calculate confidence intervals (CI). Survey weights were defined as the inverse probability of a sampled individual being included in the survey. To quantify socioeconomic status (SES), a principal components analysis was used, including variables reflecting the availability of durable household goods; the first principal component was subsequently divided into quintiles to construct the SES indicator [15]. It was assumed that data were missing completely at random; however, missing data were rare, and this assumption should have minimal impact on the results.

CI for the national and oblast-level estimates were set at 95% and constructed using a t-distribution. In contingency table analyses assessing the relationships between categorical covariates, the design-based Pearson Chi-square test was used, correcting the Chi-square statistic and basing inference on the F-distribution to account for the survey design [16]. In order to assess the relationship between the binary outcomes of interest and the covariates, which constitute a mixture of continuous, ordinal, and discrete covariates, a weighted logistic regression model with linearized robust variance estimates was used to adjust for the survey design. All hypothesis tests were two-sided and performed at the 0.05 level of significance.

## Results

In the survey, 136 SAs out of the total 199 SAs were sampled. The total sample size was 2,584 women. Descriptive statistics for the study population are shown in Table 1. Most respondents were between 20 and 39 years old, married, educated to secondary level, and of Uzbek nationality. Most of the population (78.3%) lived in a rural location. SES and urban/rural location were highly correlated, with higher SES in more urban locations (Table 1).

**Table 1:** Demographic information for the study population, women of reproductive age in Uzbekistan.

Demographic Variable	Percentage (95% CI)
Age groups	
15–19	6.8 (5.8, 8.0)
20–29	37.8 (35.6, 40.0)
30–39	37.2 (35.0, 39.4)
40–49	18.2 (16.5, 20.1)
Education	
Less than secondary	9.7 (8.3, 11.2)
Secondary	61.8 (59.3, 64.3)
College	20.1 (18.3, 22.1)
Higher	8.4 (6.9, 10.2)
Nationality	
Uzbek	87.9 (85.9, 89.7)
Tajik	4.7 (3.5, 6.2)
Kazakh	2.0 (1.5, 2.6)
Karakalpakstan	2.0 (1.4, 2.7)
Other (Russian, Tatar, Turkmen, Kyrgyz)	3.5 (2.6, 4.8)
Marital Status	
Single	15.3 (13.7, 17.0)
Married	80.8 (78.9, 82.5)
Widowed	1.3 (0.1, 2.0)
Divorced/Separated	2.6 (2.0, 3.4)
Location Setting	
Rural	78.3 (73.0, 82.9)
Urban	21.7 (17.1, 27.0)
SES quintiles – Urban	
1 (Lowest)	13.0 (11.0,15.4)
2	18.8 (16.8, 21.0)
3	24.2 (22.1, 26.4)
4	20.7 (18.7, 22.8)
5 (Highest)	23.3 (21.1, 25.7)
SES quintiles – Rural	
1 (Lowest)	53.3 (46.3, 60.1)
2	23.2 (18.9, 28.2)
3	12.5 (8.9, 17.1)
4	6.7 (4.6, 9.7)
5 (Highest)	4.3 (2.3,8.1)

### Biochemical Measures

Anemia: The estimated mean hemoglobin concentration among WRA in Uzbekistan was 121.6 g/L (CI: 120.4, 122.7), with an anemia prevalence of 34.4% (CI: 32.0, 36.7), indicating a moderate public health problem in the country [17]. There was marginal evidence that the prevalence of anemia varied within the regions of the country (p = 0.07). Surkhandarya oblast and the Republic of Karakalpakstan had the highest prevalence of anemia, estimated at 45.6% (CI 34.5, 57.2) and 44.2% (CI 36.6, 52.1), respectively. Prevalence of anemia varied by nationality (p = 0.02), with the estimated prevalence of anemia highest in respondents of Karakalpakstan nationality (49.2%) and Kazakh nationality (47.8%), compared to those of Uzbek (33.9%), Tajik (37.1%), and other nationalities (26.0%). Among women with low concentrations of hemoglobin, the majority had mild anemia (84.7%), whereas only 12.4% had moderate and 2.8% had severe anemia; estimated severity of anemia by region is shown in Table 2.

**Table 2:** Proportion (and 95% CI) of women with normal hemoglobin or anemia (classified by concentration of hemoglobin) nationally and by oblast.

Oblast	Normal (Hb^1^ 120 – 179 g/L)	Mild anemia (Hb 91 –119 g/L)	Moderate anemia (Hb 70 – 90 g/L)	Severe anemia (Hb ≤69 g/L)
Tashkent city	71.6 (63.4, 78.6)	24.5 (18.0, 32.4)	2.5 (1.0, 6.2)	1.4 (0.3, 5.9)
Tashkent oblast	65.1 (57.3, 72.2)	30.9 (23.6, 39.4)	4.0 (1.6, 9.7)	0
Syrdarya	66.4 (57.7, 74.0)	29.4 (22.4, 37.4)	3.5 (1.4, 8.3)	0.8 (0.2, 3.4)
Samarkand	67.7 (59.5, 75.0)	28.1 (20.7, 36.8)	4.0 (1.6, 9.5)	0.3 (0.0, 2.2)
Surkhandarya	54.4 (42.8, 65.5)	35.3 (26.9, 44.8)	7.4 (3.8, 14.2)	2.8 (0.8, 9.2)
Navoi	68.2 (59.9, 75.5)	23.8 (17.3, 31.7)	6.6 (3.4, 12.5)	1.4 (0.3, 6.8)
Namangan	68.1 (59.8, 75.5)	28.1 (21.3, 36.2)	3.1 (1.4, 6.8)	0.7 (0.0, 4.6)
Jizak	70.6 (62.9, 77.3)	27.2 (20.9, 34.6)	2.2 (0.9, 5.0)	0
Kashkadarya	62.7 (54.4, 70.2)	32.0 (24.9, 40.1)	3.6 (1.7, 7.4)	1.7 (0.6, 5.1)
Fergona	72.2 (63.5, 79.5)	24.1 (17.3, 32.4)	2.9 (1.2, 6.7)	1.0 (0.2, 3.4)
Khorezm	65.4 (56.7, 73.1)	25.5 (18.3, 34.4)	7.4 (4.3, 12.5)	1.7 (0.4, 6.4)
R. Karakalpakstan	55.8 (47.8, 63.4)	38.5 (31.2, 46.3)	4.7 (2.5, 8.6)	1.1 (0.3, 3.9)
Andijan	65.0 (58.1, 71.4)	28.9 (23.0, 35.7)	5.8 (2.9, 11.1)	0.3 (0.0, 1.3)
Bukhara	63.2 (55.1, 70.6)	32.8 (25.4, 41.1)	3.2 (1.5, 6.6)	0.9 (0.2,3.9)
Total (country)	65.6 (63.2, 67.9)	29.2 (27.0,31.5)	4.3 (3.4, 5.4)	1.0 (0.6, 1.5)

1 Hb  =  hemoglobin concentrations.

Prevalence of anemia varied by SES (p = 0.03), with the lowest anemia prevalence in the highest SES quintile. Prevalence of anemia was 27.8% (CI: 23.6, 32.6) in the highest quintile compared to 36.1% (CI 33.5, 38.9) in the remaining four SES quintiles (estimated prevalence was similar across these four quintiles).

Inflammation: Nationally, the mean CRP concentration was low (1.1 mg/L; CI: 1.0, 1.1), as was the prevalence of inflammation (4%; CI: 3.3 – 4.7) [14]. There were no statistically significant differences in inflammation prevalence by oblast (p = 0.68), nationality (p = 0.08), or SES (p = 0.35).

Iron status: Although there was little inflammation in the study population, ferritin concentrations were adjusted for the presence of inflammation, using the calculated factors from the meta-analysis of Thurnham *et al* [14]. The estimated overall corrected geometric mean ferritin concentration was 17.1 µg/L (CI: 16.4, 17.9) (the distribution of ferritin concentrations was highly right skewed). The prevalence of low ferritin concentrations was 47.5% (CI: 45.1, 49.9) (Table 3) [18]. Prevalence of depleted iron stores varied by oblast (p<0.0001), with the highest estimated prevalence in the Republic of Karakalpakstan (63.9%; CI: 53.7, 74.1) and the lowest in Namangan (31.8%; CI: 24.0, 39.6). Iron depletion varied by nationality (p = 0.002), with the highest prevalence (71.0%, CI: 60.1, 79.9) in Karakalpakstan.

**Table 3:** Geometric mean serum ferritin concentrations adjusted for inflammation and prevalence of depleted in stores (ferritin <12 µg/L) in women of reproductive age nationally and by oblast in Uzbekistan, 2008.

		Geometric mean serum ferritin concentrations adjusted for inflammation* (µg/L)			Depleted iron stores % Serum ferritin <12 µg/L		
	n	Crude estimate	Weighted estimate	95% CI	Crude prevalence	Weighted prevalence	95% CI
Tashkent city	171	18.4	18.0	15.2, 21.4	44.4	44.9	35.7, 54.1
Tashkent oblast	190	13.9	13.9	12.2, 15.8	57.9	58.0	48.6, 67.4
Syrdarya	171	15.0	14.4	12.3, 17.0	52.0	52.9	43.1, 62.7
Samarkand	189	19.7	21.3	17.0, 26.6	40.2	36.7	26.6, 46.8
Surkhandarya	190	20.2	20.3	17.4, 23.5	39.5	40.1	31.2, 49.0
Navoi	171	17.9	18.6	15.3, 22.6	47.4	46.0	36.3, 55.7
Namangan	170	22.8	23.1	19.8, 26.9	32.4	31.8	23.9, 39.7
Jizak	190	15.1	15.1	12.8, 17.7	51.6	51.6	43.1, 60.1
Kashkadarya	190	19.8	19.0	15.8, 22.7	41.6	43.6	34.4, 52.8
Fergona	190	14.2	15.0	12.7, 17.8	57.9	54.4	45.0, 63.8
Khorezm	152	14.6	14.2	11.9, 17.1	55.3	56.7	46.3, 67.1
R. Karakalpakstan	209	10.8	11.7	10.1, 13.6	68.9	63.9	53.7, 74.1
Andijan	209	18.6	18.3	15.9, 21.0	43.1	43.7	35.7, 51.7
Bukhara	190	14.2	14.4	12.1, 17.0	57.9	56.9	47.4, 66.4
Total (country)	2582	16.4	17.1	16.4, 17.9	49.5	47.5	45.1, 49.9

Prevalence of iron depletion was marginally associated with SES (p = 0.04). The highest prevalence of iron depletion was found in the third quintile of SES (54.2%; CI: 49.2, 59.0); and the lowest prevalence was found in the highest SES quintile (44.0%; CI: 38.8, 49.3), though this estimated prevalence was similar to the national prevalence of iron depletion (47.5%).

Folate status: The estimated mean folate concentration among WRA in Uzbekistan was 11.9 nmol/L (CI: 11.7, 12.2); and the prevalence of low folate was 28.8% (26.8, 30.8) (Table 4), with a large range by oblast (p<0.0001) from 3.0% (CI: ±3.1) deficiency in Bukhara to 45.0% (CI: 34.9, 55.1) in Navoi. There was evidence of variation in folate deficiency prevalence by nationality (p = 0.03), with the highest estimated prevalence in the Tajik nationality (39.6%; CI: 29.4, 50.8).

**Table 4:** Geometric mean folate concentrations and prevalence of low folate concentrations (<10 nmol/L) in women of reproductive age nationally or by oblast in Uzbekistan.

		Folate concentrations (nmol/L)			Prevalence of folate concentrations 10 nmol/L		
Oblast	n	Crude estimate	Weighted estimate	95% CI	Crude prevalence	Weighted prevalence	95% CI
**Tashkent city**	170	14.0	13.9	12.7, 15.2	14.7	14.1	7.2, 21.0
**Tashkent oblast**	186	11.0	11.1	10.3, 12.0	31.2	29.9	21.7, 38.1
**Syrdarya**	170	10.8	11.1	10.1, 12.2	26.5	24.1	16.3, 31.9
**Samarkand**	190	12.2	12.8	11.6, 14.1	28.9	26.8	18.7, 34.9
**Surkhandarya**	190	11.4	11.2	10.5, 12.0	35.3	37.1	28.2, 46.0
**Navoi**	169	10.5	10.3	9.6, 11.1	44.4	45.3	35.2, 55.4
**Namangan**	168	9.8	9.9	9.4, 10.4	45.8	44.4	35.6, 53.2
**Jizak**	189	10.2	10.3	9.7, 11.0	42.9	41.0	32.0, 50.0
**Kashkadarya**	190	11.3	11.2	10.4, 12.2	28.4	27.4	19.6, 35.2
**Fergona**	189	9.8	9.8	9.3, 10.3	54.5	53.9	45.5, 62.3
**Khorezm**	151	13.4	13.4	11.9, 15.1	16.6	16.8	9.7, 23.9
**R. Karakalpakstan**	206	12.1	12.3	11.2, 13.5	21.4	21.1	14.3, 27.9
**Andijan**	207	13.2	12.9	11.8, 14.1	14.0	15.2	9.5, 20.9
**Bukhara**	188	18.8	18.7	17.7, 19.8	3.7	3.4	0.3, 6.5
**Total (country)**	2563	11.9	11.9	11.7, 12.2	29.1	28.8	26.8, 30.8

SES was associated with folate deficiency (p = 0.001). Estimated prevalence of folate deficiency increased monotonically with SES quintile. The prevalence of women with folate deficiency in the lowest SES category was 36.2% (CI: 31.3, 41.4) and decreased to 22.4% (CI: 18.6, 27.7) in the highest SES category.

### Relationship between anemia, folate deficiency, and iron depletion

Iron depletion and folate deficiency were both associated with anemia (p<0.0001 for both). Estimated anemia prevalence was 20.2% (CI: 17.6, 23.2) among WRA without iron depletion; and 45.8% (CI: 42.5, 49.2) among WRA with iron depletion. Estimated anemia prevalence was 24.7% (CI: 22.4, 27.1) among WRA without folate deficiency and 51.3% (CI: 46.9, 55.8) among WRA with folate deficiency. Folate deficiency and iron depletion were also both associated with severity of anemia (p<0.0001).

To investigate the relative importance of folate deficiency and iron depletion in anemia severity, a four-category indicator was constructed, with categories: no iron or folate depletion; iron depletion with no folate depletion; folate deficiency with no iron depletion; and both iron and folate depletion. Anemia severity is examined within levels of this indicator in Table 5. Anemia severity is highly associated with this indicator (p<0.0001); WRA with iron and folate depletion have the most severe anemia, followed by women with folate deficiency and no iron depletion. Restricting to women with either folate or iron depletion, but not both, anemia prevalence was similar between the folate and iron depleted groups, but folate deficiency was associated with more severe anemia than iron depletion (p<0.0001).

**Table 5:** Prevalence of anemia by stage within levels of iron and folate deficiency (95% CI). The p-value testing no difference in anemia stage by ferritin/folate deficiency is <0.0001.

Anemia Stage	Normal (>120 g/L)	Mild (91-120 g/L)	Moderate (70–90 g/L)	Severe (≤69 g/L)
Iron and folate status				
Adequate ferritin and folate	84.8 (82.0, 87.3)	14.6 (12.2, 17.4)	0.6 (0.2, 1.7)	0
Adequate ferritin, low folate	59.0 (52.3, 65.4)	32.2 (26.2, 38.8)	7.0 (4.4, 10.9)	1.9 (0.7, 4.9)
Low ferritin, adequate folate	58.8 (54.7, 62.7)	40.2 (36.3, 44.2)	0.8 (0.4, 01.6)	0.2 (0.0, 0.7)
Low ferritin and low folate	36.1 (30.1, 42.5)	41.4 (35.4, 47.6)	18.3 (14.1, 23.5)	4.2 (2.5, 7.0)
Total	65.5 (63.1, 67.8)	29.3 (27.1, 31.6)	4.3 (3.4, 5.4)	1.0 (0.6, 1.6)

**Figure 1:**
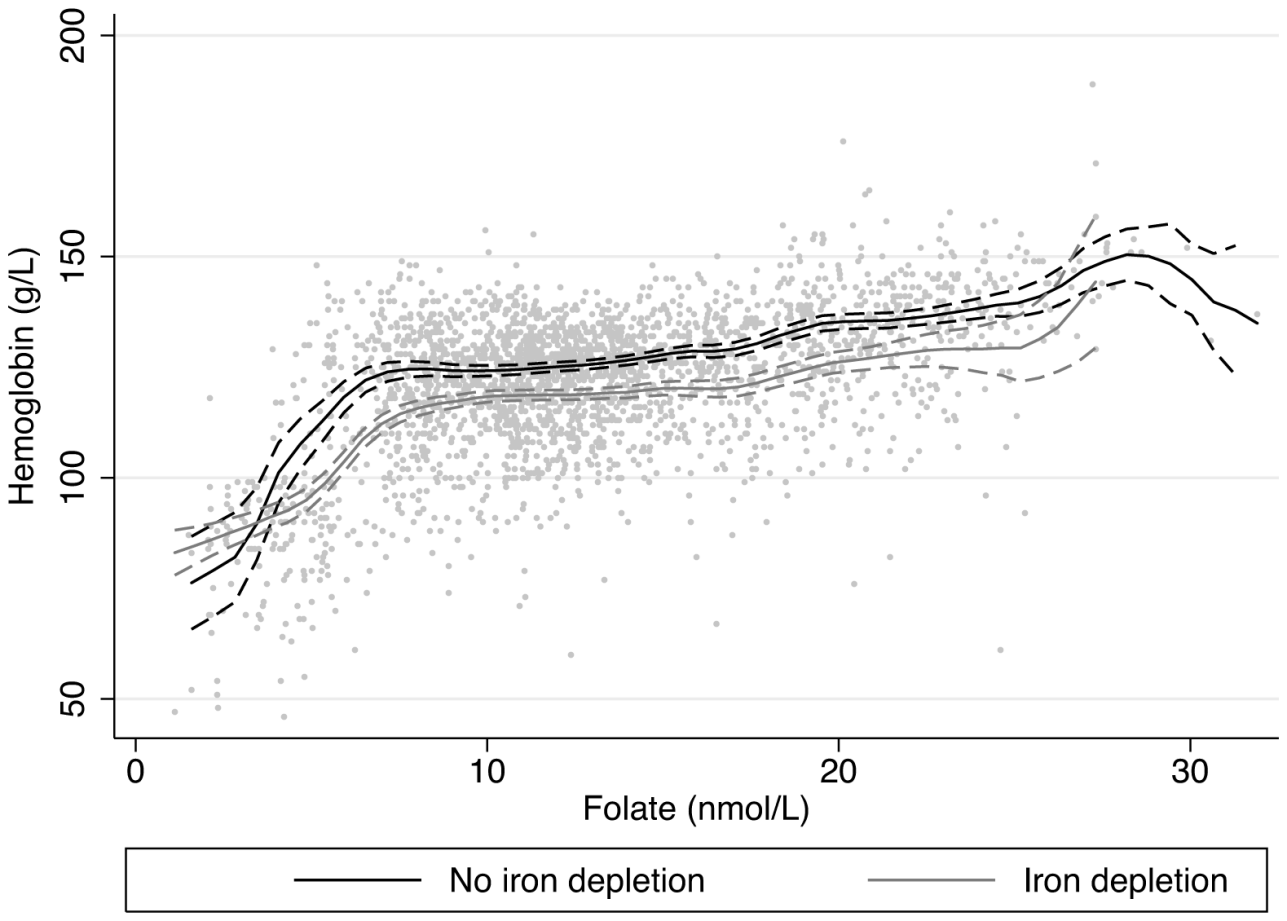
Average hemoglobin concentration as a function of folate concentrations stratifying by iron depletion with corresponding 95% confidence intervals. –––––– No iron depletion, –––––– Iron depletion.

**Figure 2:**
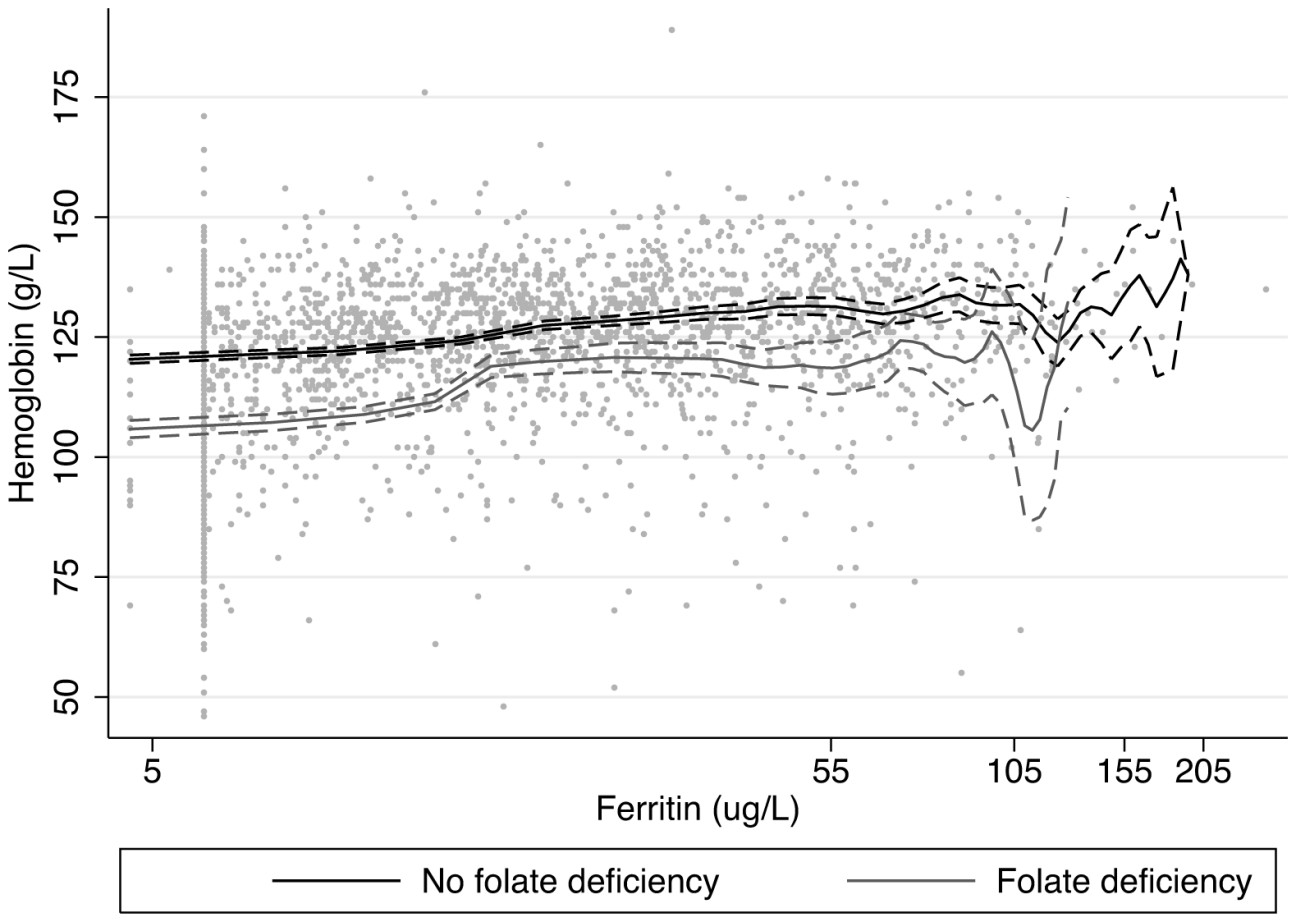
Average hemoglobin concetration as a function of adjusted ferritin concentrations, stratifying by folate deficiency with corresponding 95% confidence intervals. –––––– No folate depletion, –––––– Folate depletion.

In Figure 1, average hemoglobin is non-parametrically estimated using kernel weighted local polynomial smoothing, with corresponding 95% confidence intervals. Hemoglobin concentrations are plotted as a function of folate concentrations, stratifying by iron depletion; and as a function of ferritin concentrations, stratifying by folate deficiency (Figure 2). Average hemoglobin concentrations do not drop far below 120 g/L among those with adequate folate status; among those with folate deficiency, average hemoglobin does not reach 120 g/L until ferritin concentrations are approximately 17 µg/L. Average hemoglobin concentrations steeply increase as a function of folate concentrations, when folate levels are <10 nmol/L. Average hemoglobin concentrations surpass 120 g/L when folate concentrations are approximately 7 nmol/L for WRA without iron depletion, and approximately 15 nmol/L among WRA with iron depletion.

#### Availability of UDM first grade flour or the grey loaf

Nationally, 96.9% (CI: 96.0, 97.5) of WRA had some type of wheat flour in the household. UDM first grade flour was identified in 59.8% (CI 56.8, 62.8) of households, with substantially lower percentages in Tashkent city (2.1%; CI: 0.1, 6.1) and Tashkent oblast (32.4%; CI: 19.9, 48.1). In 88 cases (3.5%), respondents were not able to identify the type of the flour at their households, as often the flour was purchased in packages without labels.

The grey loaf was less popular than UDM flour, with only 11.5% (CI: 9.7, 13.3) of households having the grey loaf at the time of the survey. However, in Tashkent city and Tashkent oblast, 72.1% (CI: 63.8, 80.4) and 38.5% (95%CI: 24.6, 52.4) of households respectively had the grey loaf in the household. Altogether, 71.3% (CI: 68.7, 73.9) of households consumed either the grey loaf or home-baked bread made from UDM first grade flour the day of the survey. Consumption of fortified products varied by oblast (p = 0.001), with the lowest estimated proportion reported in Jizak (53.2; CI: 44.7, 61.6) and the highest in Navoi (82.7; CI: 74.9, 88.5).

The qualitative iron spot test carried out at the household identified that 41.6% (CI: 39.2, 43.9) of all flour tested was fortified. Fortification rates varied by oblast (p<0.001) with the highest estimates of 73.5% (CI: 65.5, 80.2) in Bukhara and the lowest in Syrdarya oblast (10.1%; 95%CI: 5.6, 17.6) and Tashkent city (11.4%; 95%CI: 7.2, 17.6).

UDM first grade flour was found more frequently in households with lower SES (p<0.001). In the lowest SES group, 65.4% (CI: 60.1, 70.3) had UDM first grade flour, compared to 45.0% (CI: 40.0, 50.2) in the highest group. In urban areas, UDM first grade flour was available in 42.0% (CI: 33.7, 50.8) of households compared to 62.6% (CI: 59.2, 66.0) in rural areas (p = 0.001).

A multivariate logistic regression model was used to further examine predictors of the availability of first grade flour. The following covariates were included in the regression model: age, education, SES, urban/rural location, knowledge that UDM flour is fortified, whether the woman bakes her own bread, anemia history, and iron use history. Education was not statistically significant in the regression model, or in an unadjusted analysis, and was dropped from the regression model. Age was not a statistically significant predictor (p = 0.08), but was included in the final model, as it might be an important confounder. SES was included as a linear term in the model. An interaction between urban/rural location and SES was included in the final regression model, to examine effect modification of the relationship between SES and availability of UDM first grade flour by location. The conditional odds ratios from this regression model were examined to help understand predictors of using UDM flour.

The strongest predictor of having UDM first grade flour was whether or not a woman bakes her own bread (OR: 3.84; CI: 2.53, 5.82; p<0.001). Knowledge that UDM flour is fortified was associated with an increased likelihood of having the flour in the household (OR 1.28; CI: 1.06, 1.56; p = 0.01). Women with a history of anemia were slightly less likely to have first grade UDM flour in the house than those with no history (OR: 0.81; CI: 0.65, 1.01; p = 0.06), though this result was not statistically significant. Women who had taken iron supplements within the last two years (OR: 1.29, CI: 0.96, 1.72) and those who had taken iron tablets more than two years ago (OR: 1.16, CI: 0.89, 1.51) were slightly more likely to have UDM first grade flour in the household than those who had never taken iron, but this result was also not statistically significant (p = 0.23).

Among rural women, there was no strong evidence of an association between SES and having flour in the household (OR for a one quintile decrease in SES: 1.02, CI 0.94, 1.10; p = 0.61). Among urban women, decreasing SES was associated with an increase in UDM first grade flour possession (OR for a one quintile decrease in SES: 1.36, CI 1.12, 1.66; p = 0.003).

### Relationship between biochemical measures and UDM flour

There were no statistically significant differences in anemia prevalence or iron depletion prevalence by possession of UDM flour (p = 0.58 anemia; p = 0.29 iron depletion) or possession of grey loaf (p = 0.26 anemia; p = 0.30 iron depletion).

Those who had a grey loaf in the household the day before the survey had a significantly lower (p = 0.03) prevalence of folate deficiency, with an estimated prevalence 22.7% (CI: 17.6, 28.8) among those with grey loaf compared to 29.7% (CI: 27.6, 31.9) among those without the grey loaf. Those who had UDM flour in the household had a higher prevalence of folate deficiency (p = 0.03), with an estimated prevalence of 26.2% (CI: 23.4, 29.2) among those without UDM flour compared to 30.8% (CI: 28.0, 33.8) among those with UDM flour. However, UDM and grey loaf possession are both highly correlated with urban/rural location and socioeconomic status; the associations between UDM/grey loaf possession and folate deficiency were not statistically significant in a multivariate logistic regression analysis adjusting for SES or urban/rural location. Consequently, the decrease in low folate prevalence should not be directly attributed to grey loaf possession or the increase in low folate prevalence to possession of UDM flour

#### Knowledge

Among WRA in Uzbekistan, 54.5% (CI: 52.2, 56.8) reported that they had heard of fortified food. Nationally, 47.0% (CI: 44.6, 49.4) of respondents mentioned first grade UDM flour or the grey loaf among the types of fortified food. Among WRA, 32.1% (CI: 29.9, 34.3) were aware that first grade UDM flour or the grey loaf are fortified with iron. However, only 3.7% (CI: 2.8, 4.6) of respondents were able to mention the benefits of fortified flour/bread consumption, and only 12.2% (CI: 10.7, 13.9) of respondents were aware how to distinguish fortified flour from non-fortified flour. The “non-polvon” logo (healthy food logo) for fortification was recognized by 36.9% (CI: 34.4, 39.4) of women; however, only 23.1% (95%CI: 21.0, 25.2) of respondents understood the meaning of this healthy food logo. The level of knowledge of the women about IDA was low, as only 12.5% (CI: 11.0, 14.0) of women were able to identify IDA causes (e.g. not eating enough meat, vegetables and fruit and drinking tea/coffee with meals) and 17.4% (CI: 15.4, 19.4) knew who the most-at-risk populations were. Only 41.9% (CI: 39.5, 44.3) of WRA were able to list at least three signs or consequences of anemia, and 5.9% (CI: 4.6, 7.2) of the women were aware how to avoid IDA.

#### Consumption of heme iron-containing food

The estimated proportion of WRA in Uzbekistan who ate heme iron-rich foods (poultry, meat, fish or eggs) at least twice in the last week was 96.2% (CI: 95.1, 97.1); and at least once the day before the survey was 91.4%; (CI: 90.0, 92.8). Consumption of enhancers of iron absorption (green leaves, vegetables or fruits) was also high, with 90.2% (CI: 88.7, 91.7) consuming these foods at least twice in the week prior to the survey; and 97.1% (CI: 96.3, 98.0) consuming these foods the day before the survey. However, consumption of iron absorption inhibitors during a meal or directly after a meal was also high, with 94.7% (CI: 93.5, 95.8) of women reporting drinking coffee or tea with a meal the day before the survey.

To assess the impact of dietary choices on the biochemical measures, the consumption of iron-rich foods at least twice in the week preceding the survey was used as a proxy for consumption of heme iron containing foods in general, as this indicator should reflect regular consumption of heme rich foods. There was no evidence of an association between consumption of heme rich foods and iron depletion (p = 0.29) or anemia (p = 0.68); only 97 women (3.8%) did not regularly consume heme rich foods. The estimated prevalence of low ferritin in those who ate ‘heme iron-rich foods’ foods was 47.8% (CI: 45.3, 50.2) compared to 41.4% (CI: 30.6, 53.0) in those who did not; and the prevalence of anemia was 34.4% (CI: 32.1, 36.9) in those who ate the foods versus 32.1% (CI: 22.2, 43.8) in those who did not eat the foods.

There was marginal evidence of an association between low folate concentrations and consuming heme iron-rich foods (p = 0.07); the prevalence of low folate concentrations in those who ate heme iron-rich foods was 28.5% (CI: 26.4, 30.6), compared to 39.1% (CI: 28.0, 51.6) in those who did not. When adjusted for relevant confounders (age, SES, anemia history, education) in a multivariate analysis, this association was attenuated and not statistically significant (p = 0.18).

## Discussion

In this paper, coverage of the flour fortification program and biochemical measures of folate deficiency, iron deficiency, and anemia among WRA are described following the implementation of a national flour fortification program. By measuring the biochemical measures, alongside coverage of fortified products and knowledge of dietary and health concepts, areas with potential for improvement were identified. The LC-LQAS survey was carried out three years after the initiation of the NFFP, in order to give sufficient time for the fortification program to have an impact on the target population group, WRA.

### 

#### Biochemical results

The aim of reducing the prevalence of anemia in non-pregnant WRA to 48% was surpassed, as anemia prevalence was estimated at 34.4% (CI: 32.0, 36.7). Although evaluating the direct impact of the fortification program is difficult, it is likely that the UDM first grade flour and grey loaf fortification project contributed to this decline in anemia prevalence. However, the interpretation of the biochemical results might also be somewhat compromised, as the survey coincided with the time the premix was in short supply.

The national survey results were compared to the sentinel-site data based on 40 households collected between 2004 and 2007. Results from the sentinel study should be interpreted with caution, as they were not nationally representative. Nevertheless, between 2004 and 2007, estimates of anemia prevalence decreased by 23.5% (from 51.3% to 27.8%), the 2007 result being comparable to that obtained in the LC-LQAS survey (34.4%) reported here. Confidence intervals for the sentinel study were not available, but the decline was not statistically significant. The sentinel site study and the LC-LQAS survey cover the time period of the NFFP and suggest a positive effect of the fortification program on anemia prevalence.

The estimated overall prevalence of depleted iron stores (ferritin<15 µg/L) increased from 28.2% in 2004 to 33.3% in 2007 in the sentinel study and was highest in the LC-LQAS survey (47.5%). The higher prevalence of depleted iron stores in the LC-LQAS survey was likely due to the inclusion of a representative sample from all regions of the country, including the poorer regions. In the Republic of Karakalpakstan, the prevalence of low ferritin was the highest in the country (63.9%). Sub-clinical inflammation, indicated by elevated concentrations of the acute phase proteins, could impact on serum ferritin concentrations causing them to increase, thus underestimating depleted iron stores [19]. Based on CRP concentrations, little inflammation was found among the women; however, underlying chronic inflammation might be more of a problem than acute inflammation in Uzbekistan. Raised α-1 acid glycoprotein (AGP) concentrations would indicate any chronic inflammation [14], but it was not possible to measure AGP in this survey.

In Uzbekistan, the UDM first grade flour is often mixed with other flours during bread making, diluting the fortification effect; a more bioavailable form of iron might help improve the biological impact. Later versions of the KAP premix have used more bioavailable sources of iron, such as NaFeEDTA or ferrous sulfate and are an option, which could be considered.

In the LC-LQAS survey, an estimated 29.1% of women were folate deficient, similar to the prevalence obtained in 2004 (33.3%) and 2007 (24.1%) from the sentinel study. Folate deficiency among WRA is likely driven by low folate intake in the diet or cooking processes that destroy folate. Consistent consumption of adequately fortified flour should improve folate concentrations in WRA. The prevalence of folate deficiency varied significantly by oblast and may be explained by an iron-folic acid supplementation program, distributing weekly tablets to WRA in some oblasts at the time of the survey; however, there is no data on which oblasts continued to get the supplements.

Folate and iron deficiency were strongly associated with stage of anemia. While women with both iron and folate deficiency had the most severe anemia, folate deficiency was associated with more severe anemia than ferritin deficiency, suggesting that folate was a limiting nutrient in iron nutrition. Folate is needed for the formation of hemoglobin, and deficiency impairs the maturation of young red blood cells, resulting in anemia [20]. Prioritizing folate over iron intake may have more of an impact on anemia prevalence in this population, as average hemoglobin concentrations did not reach 120 g/L until folate concentrations were >10 nmol/L; where folate deficiency was present, only higher concentrations of ferritin (17 µg/L) were associated with hemoglobin concentrations of 120 g/L. Both iron and folate are lacking in the diet among WRA. While the fortification program has probably contributed to the reduction in anemia prevalence in the population, there is still need for further effort to improve dietary intake of iron and folate. Increased availability of nutritional supplements and improved fortification efforts could help increase plasma folate and ferritin concentrations and lower anemia prevalence. Additionally, the monotonic relationship between SES and folate deficiency prevalence suggests a need to ensure that nutritional knowledge and interventions reach rural, low SES women.

#### Availability of fortified flour and bread

Overall, the flour fortification program was implemented successfully, with fortified flour available throughout Uzbekistan. At the time of the survey, 59.8% (CI: 56.8, 62.8) of households reported having UDM first grade wheat flour or bread made from the flour. The fortified grey loaf was more popular than UDM flour in the urban locations of Tashkent City and Tashkent Oblast where there are more bakeries selling ready-made bread. Combining the data together, 71.3% of households (CI: 68.7, 73.9) had either a grey loaf or home-baked bread made from UDM first grade flour on the day of the survey, which is close to the program target of 80% of households.

UDM first grade flour coverage varied geographically and was highest among low SES and rural women. This flour is perceived as being of poor quality, and those who can afford the more expensive non-fortified Kazakh flour will purchase it. Conversely, in areas where there are higher rates of poverty, such as the semi-autonomous Republic of Karakalpakstan, households largely consume flour that they produce and mill themselves (non-fortified); rates of anemia and depleted iron stores were higher among women in these regions relative to the rest of Uzbekistan. No statistically significant associations were observed between anemia or iron depletion prevalence and possession of fortified products. Associations between possession of fortified products and folate deficiency may be attributable to socioeconomic and location differences between those with and without the fortified products.

In early 2008, there was a sharp drop in fortified first grade flour milled due to a shortage of fortificant. Premix supplies were available again in June 2008, but it was not until later in 2008 that production once again reached >90% of total UDM first grade flour milled (personal communication). The iron spot test found 41.6% (CI: 39.2, 43.9) of the flour was adequately fortified, and the fortification program target of 30% non-pregnant WRA regularly consuming fortified wheat flour was achieved. However, there was substantial variability by oblast in the percentage of adequately fortified flour samples. While UDM has flour-mills in all oblasts, the availability of premix, as well as the quality of the fortification process, may vary across mills. An independent end-of-project evaluation was carried out in 2009, subsequent to the survey and the premix shortage. This evaluation reported that 46% of iron spot tests at mills showed adequate fortification [5], suggesting that more mill-level monitoring by external quality control laboratories is needed. It was not possible to assess the adequacy of the grey loaf fortification, as there is no simple field qualitative test for bread fortification; the flour used in grey loaf is likely from the same mills, suggesting that the fortification pattern would be similar.

#### Knowledge of fortification and anemia

Knowledge about fortified foods and the fortification project was low among women. While the healthy food logo was recognized by 36.9% of WRA, only 23.1% of women understood what the logo represented and only 12.5% of WRA knew how to distinguish fortified flour from non-fortified flour. Measuring whether target groups had seen the logo says little about how WRA link this logo to fortified food or how the logo might affect food purchasing or consumption habits. The success of the fortification program requires that women understand and accept that fortified flour improves their health. The survey results suggest that communication messages for fortification might need to move from logo recognition to include a larger component of consumer nutrition education.

Less than half of the women interviewed knew the signs and consequences of IDA, and even fewer knew the causes of IDA. As with the logo recognition, more widespread health education is needed at opportune times, e.g. such as visiting SVP/policlinics or in schools for girls and distributing nutritional information to both women and girls.

#### Dietary patterns

The diet of the women consisted of foods that can reduce the risk of anemia, including iron-rich foods, such as meat, fish or poultry, and iron-enhancers such as vegetables and fruits. Data on food frequency from the 2002 Health and Examination Survey suggest that red meat was eaten by women on average 5 times per week, but rural (3.9 days)/urban (7 days) differences existed [2]. Similarly, consumption of dark green vegetables differed by location, as these foods were eaten 7 days/week in urban and 4.7 days/week in rural areas; fresh fruit was eaten daily in both locations. On the other hand, women drank tea or coffee with most meals, reducing the bioavailability of dietary iron. The relationship between diet and anemia prevalence is complex. The amount of dietary iron absorbed is mainly determined by the body stores of iron and the iron content and bioavailability of the diet [8]. In populations, such as Uzbekistan, with low meat (once/day) and high phytate or iron-binding phenolic compound intake (tea/coffee with meals), the bioavailability of iron from the diet is estimated at about 10%. In such a population, approximately 40 – 50% of the women will have no iron stores (8). The estimates from the Uzbekistan 2008 survey fall into this expected range, as 47.5% of women had depleted iron stores. It is probable that most women cannot afford to eat more expensive heme-rich foods, but efforts in the country to educate women not to drink tea at meal times to reduce the high phytate intake and improve the bioavailability of the iron at meal times are underway.

#### Conclusions and subsequent actions

The fortification of first grade UDM wheat flour with a variety of micronutrients, including iron and folic acid, coincided with a substantial decline in the prevalence of anemia in WRA in Uzbekistan. But the high prevalence of depleted iron stores suggests that women are still not eating enough iron in the diet or are drinking too much tea at meal times and inhibiting iron absorption. However, prioritizing folate over iron intake may have more of an impact on anemia prevalence in this population.

The government now recommends intake of a weekly folic acid and iron tablet for WRA, children and adolescents to ensure an adequate supply of folate and iron. For non-pregnant women and adolescents, the tablet contains 350–500 µg of folic acid and 60 mg iron; and for pregnant women, 700–1000 µg folic acid and 120 mg iron. Infants <1 year are given a smaller dose of iron (30 mg). This governmental action should help decrease the prevalence of observed folate and ferritin deficiencies, and subsequently anemia.

The data from the 2008 LC-LQAS survey showed that use of fortified flour was common throughout the country. However, a higher proportion of UDM flour needed to be fortified to the appropriate level and fortification levels adequately monitored. To address this issue, mandatory fortification legislation was enacted in January 2011; this legislation stipulated that mills, both state-run and private, must meet fortification standards set for first grade flour in order to receive annual production permits. Further, in June 2011, a Presidential decree stipulated the specific vitamin-mineral mixture for the enrichment of bread making wheat flour, and stated that the flour must contain 1 mg folic acid and 33.3 g iron/100 g wheat flour (O'z DSt1098: 2011). Additionally, the decree stipulates that 1.3 mg thiamin, 2.0 mg riboflavin, 6.7 mg niacin and 14.7 mg zinc/100 g wheat flour should be added. Once flour is consistently fortified at an adequate level, this decree should also help address the observed folate and ferritin deficiencies.

To address the need for better monitoring of the program, the two laboratories responsible for external quality assurance of the fortified flour have been provided with all equipment and training needed to ensure the quality control of the fortification process. However, unfortified flour from Central Asia continues to be imported, diluting the fortification effect. A regional approach to domestic and imported flour legislation, coupled with common standards on fortification dosage and quality, would likely achieve the coverage of fortified flour consumption to produce sustainable health improvements.

The data from the survey suggested communication messages about the fortification project were not reaching the majority of women, and women's understanding of the causes of IDA was poor. Since 2008, customization of social marketing activities targeted at different population groups, e.g. consumers, producers (millers and bakers) and the media, have been implemented as an innovative and effective approach. For example, the packaging standards mandate that producers print the logo “Enriched with Iron” on flour packages and education of millers and bakers on the health implications of micronutrient deficiencies have been used to build awareness.
